# Myeloid-Derived Suppressor Cells: Ductile Targets in Disease

**DOI:** 10.3389/fimmu.2019.00949

**Published:** 2019-05-03

**Authors:** Francesca Maria Consonni, Chiara Porta, Arianna Marino, Chiara Pandolfo, Silvia Mola, Augusto Bleve, Antonio Sica

**Affiliations:** ^1^Humanitas Clinical and Research Center, Rozzano, Italy; ^2^Department of Pharmaceutical Sciences, Università del Piemonte Orientale “Amedeo Avogadro”, Novara, Italy; ^3^Center for Translational Research on Autoimmune and Allergic Diseases (CAAD), University of Piemonte Orientale, Novara, Italy

**Keywords:** emergency myelopoiesis, myeloid-derived suppressor cells (MDSCs), immunosuppression, cancer, autoimmune diseases

## Abstract

Myeloid-derived suppressor cells (MDSCs) represent a heterogeneous population of immature myeloid cells with major regulatory functions and rise during pathological conditions, including cancer, infections and autoimmune conditions. MDSC expansion is generally linked to inflammatory processes that emerge in response to stable immunological stress, which alter both magnitude and quality of the myelopoietic output. Inability to reinstate physiological myelopoiesis would fall in an “emergency state” that perpetually reprograms myeloid cells toward suppressive functions. While differentiation and reprogramming of myeloid cells toward an immunosuppressive phenotype can be considered the result of a multistep process that originates in the bone marrow and culminates in the tumor microenvironment, the identification of its driving events may offer potential therapeutic approaches in different pathologies. Indeed, whereas expansion of MDSCs, in both murine and human tumor bearers, results in reduced immune surveillance and antitumor cytotoxicity, placing an obstacle to the effectiveness of anticancer therapies, adoptive transfer of MDSCs has shown therapeutic benefits in autoimmune disorders. Here, we describe relevant mechanisms of myeloid cell reprogramming leading to generation of suppressive MDSCs and discuss their therapeutic ductility in disease.

## Introduction

Immunologic stress, such as infection and cancer, modifies the magnitude and composition of the hematopoietic output, a feature of immune regulation defined as “emergency” hematopoiesis, to guarantee proper supply of both lymphoid and myeloid cells to increased demand (1). Under steady-state conditions, myelopoiesis is a strictly regulated process that consists of a series of cell lineage commitments, encompassing sequential steps of differentiation that govern the transition of hematopoietic stem cells (HSCs) to myeloid precursors and then to mature immune cells, which is necessary to maintain the physiological levels of circulating neutrophils and monocytes (2). This highly coordinated process is orchestrated by cytokines and growth factors, which act through activation of specific transcription factors that differentially drive terminal differentiation of myeloid cells. In particular, whereas C/EBPα appears to be a major regulator of “steady-state” granulopoiesis (3), C/EBPβ (4) and Signal Transducer and Activator of Transcription 3 (STAT3) (5) promote expansion and maturation of neutrophils in emergency conditions. Moreover, interleukin-17A (IL-17A) promotes both granulocyte-colony stimulating factor (G-CSF)- and stem-cell-factor-mediated neutrophilia (6) and supports G-CSF-driven “emergency” myelopoiesis (7). Terminal macrophage differentiation is instead induced by macrophage-CSF (M-CSF) through activation of the transcription factors PU.1 and IRF8 (8). We recently showed that the retinoic acid-related orphan receptor (RORC1/RORγ) orchestrates emergency myelopoiesis by suppressing negative (Socs3 and Bcl3) and promoting positive (C/EBPβ) regulators of granulopoiesis, as well as the key transcriptional mediators of myeloid progenitor commitment and differentiation to the monocytic/macrophage lineage (IRF8 and PU.1) (9). Of note, expansion of circulating RORC1^+^ myeloid cells marked advanced cancer-related inflammation and the expansion of immature suppressive cells (9).

In acute inflammation, notably during acute infections, myeloid progenitors expand and differentiate into activated pro-inflammatory monocytes, which eventually migrate into tissues where they differentiate into macrophages and dendritic cells (10, 11). On the other hand, in chronic inflammatory states (e.g., cancer, chronic infection and autoimmune disease) the differentiation of myeloid progenitors into mature immune cells is impaired, a condition that leads to the expansion and accumulation of a population of immature myeloid cells named myeloid-derived suppressor cells (MDSCs) (12). MDSCs consist of a heterogeneous population characterized by high plasticity and strong capacity to reduce cytotoxic functions of T and NK cells (13). MDSCs are conventionally divided into 2 subsets, monocytic (M-MDSCs) and granulocytic (PMN-MDSCs), based on the expression of specific markers that differ among human and mouse cells. In humans, the M-MDSC and PMN-MDCS subsets are defined as CD11b^+^CD14^+^HLA-DR^−/low^CD15^−^ and CD11b^+^CD14^−^CD15^+^HLA-DR^low/−^, respectively, while their corresponding murine subsets are indicated as CD11b^+^Ly6C^high^Ly6G^−^ and CD11b^+^Ly6C^low^Ly6G^+^ cells (11).

From a biochemical and functional perspective, suppressive PMN-MDSCs are characterized by the production of reactive oxygen species (ROS) and arginase 1 (Arg1), whereas M-MDSCs predominantly express the inducible nitric oxide synthase (iNOS) gene and produce nitric oxide (NO). Both pathways promote depletion of the amino acid l-arginine and down-regulation of T cell receptor (TCR) ζ-chain expression, consequently leading to cell cycle arrest (14). Combined production of ROS and NO results in peroxynitration of TCR and promotes T cell apoptosis (15). Additionally, expression of indoleamine 2,3 dioxygenase (IDO) (16), cyclooxygenase (COX1) (17) and the programmed death-ligand 1 (PD-L1) (18) by activated MDSCs concur to immune suppression. MDSCs further promote T regulatory (Treg) cell expansion to prevent anti-tumor T cell effector functions (11, 19–21). A recent meta-analysis performed on a cohort of 1864 patients evaluated the prognostic value of MDSCs in various types of cancers and concluded that their elevated frequency is associated with shorter overall survival (OS) and poor disease-free survival/recurrence-free survival (DFS/RFS) (22). Based on their critical pro-tumor role, efforts are underway to define strategies that can reprogram or functionally deplete MDSCs in order to evaluate their antitumor efficacy alone or in combination with anti-checkpoint inhibitors (ICIs) (23). Since persistent immunological stress promotes the pathological differentiation of myeloid cells, MDSC expansion has been reported also in autoimmune diseases (AD) (12). Similarly, in some infections, caused either by bacteria (e.g., *M. tuberculosis, Staphylococcus aureus*) or viruses (e.g., hepatitis B virus/HBV, hepatitis C virus/HCV, human immunodeficiency viruses/HIV), the host's immune response is not able to remove the pathogen, which instead persists and leads to a chronic inflammatory state. In these pathological conditions, the accumulation of M-MDSCs is stimulated to restrict T cell effector functions and to recruit Treg cells in order to resolve inflammation and re-establish immune homeostasis (24, 25). In infections, pathogen recognition by innate immune receptors (e.g., Toll-like receptor), other than cytokines and growth factors, is the key event responsible for M-MDSCs expansion (25).

Targeting MDSCs appears to provide a specular perspective in cancer vs. autoimmune conditions. Here we discuss the role of MDSCs in cancer and autoimmune diseases, highlighting their main suppressor mechanisms and possible therapeutic interventions.

## The Immunosuppressive Armament of MDSCs and Its Impact in Cancer

**Figure 1:**
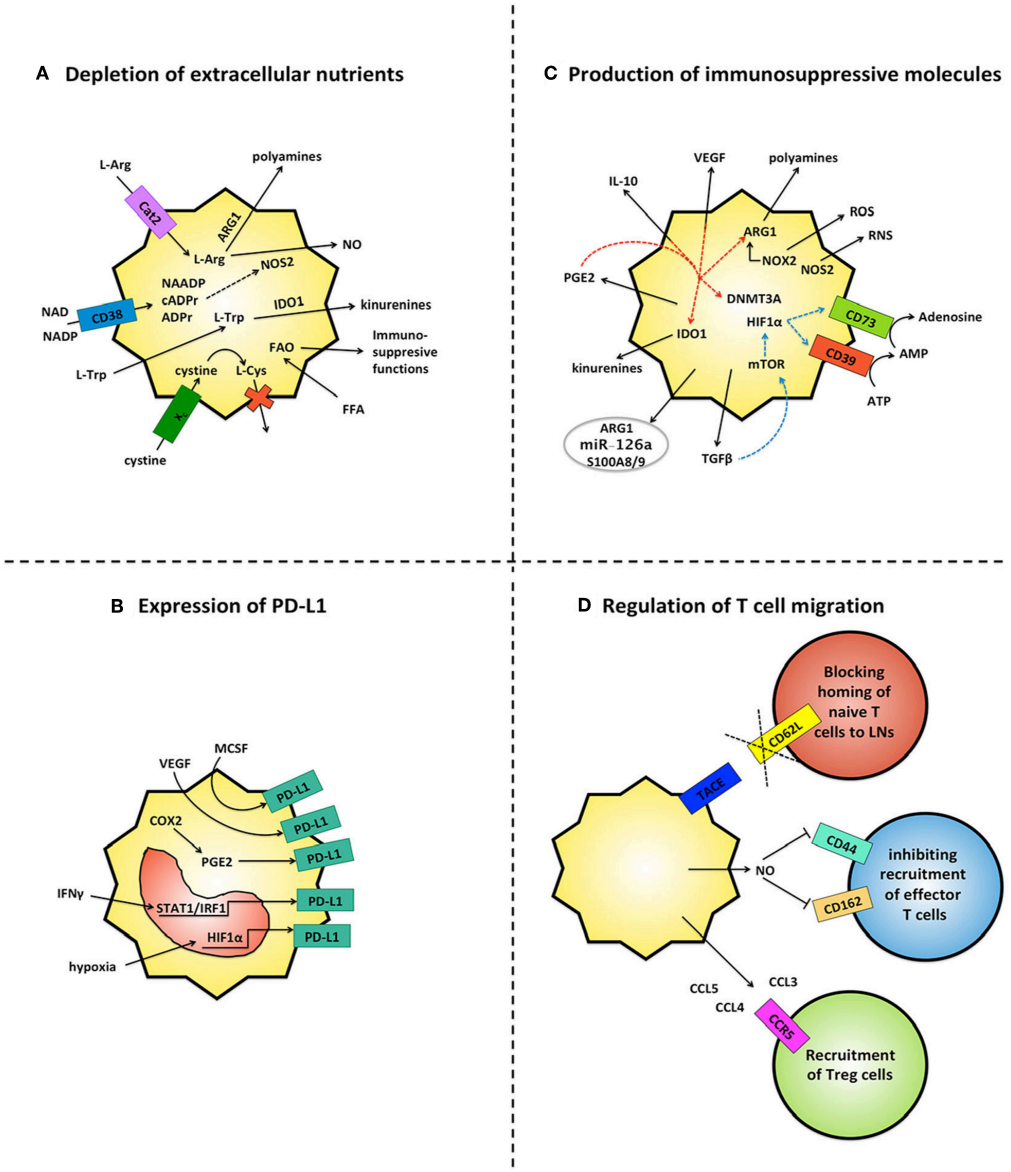
MDSCs inhibit immune responses by multiple mechanisms. **(A)** MDSCs deplete the extracellular microenvironment of essential nutrients for T cells. Through the up-regulation of metabolic enzymes (e.g. ARG1, NOS2, IDO1) and ectoenzymes (e.g. CD38) MDSCs consume copious amounts of amino acids (L-Arg, L-Trp) and NAD, and concomitantly produce molecules endowed with immunomodulatory activities (e.g. nitric oxide/NO, polyamines and kinurenines). Further, MDSCs internalize cystine without releasing the oxidized L-Cys and up-take of FFA, which fuels FAO and expression of immunosuppressive activities. **(B)** MDSCs up-regulate PD-L1 in response to multiple microenvironmental signals, including hypoxia via HIF1α, IFNγ via STAT1/IRF1, MCSF and VEGF via unknown mechanisms. Up-regulation of COX2 and PGE2 are also found associated with PD-L1 expression. **(C)** MDSCs release a range of immunosuppressive soluble molecules. They produce ROS and RNS through NOX-2 and NOS2, adenosine via CD39 and CD73, kinurenines via IDO1, polyamines via ARG1, anti-inflammatory cytokines (IL-10, TGFβ) and PGE2. Both TGFβ (blue lines) and PGE2 (red lines) also create autocrine loops that sustain the production of additional suppressive molecules. TGFβ induces the ectoenzymes CD39 and CD73 via HIF-1 α and PGE2 promotes expression of immunosuppressive molecules (IDO1, IL-10, ARG1 and VEGF) as well as repression of immunogenic-associated genes via DNMT3A. MDSCs also secrete exosomes which contain different molecules, such as immunosuppressive ARG1, inflammatory S100A8/9 and the oncogenic miR-126a. **(D)** MDSCs modulate T cell trafficking. They limit homing of naïve T cells to LNs by TACE-mediated cleavage of CD62L on T cells and they impair extravasation of effector T cells through NO-mediated down-regulation of adhesion molecules CD162 and CD44. In contrast MDSCs support the recruitment of CCR5^+^ Treg cells by production of CCL3, CCL4, CCL5.

Beyond being highly heterogeneous, MDSCs are also highly plastic (26), therefore the surrounding microenvironment shapes MDSCs' functions to suppress immune responses through multiple mechanisms (Figure 1), including depletion of metabolites critical for T cell functions, expression of immune checkpoint inhibitors, secretion of immunosuppressive molecules, production of reactive oxygen and nitrogen species and regulation of lymphocyte homing.

### Depletion of Metabolites Critical for T Cell Functions

A metabolic feature of MDSCs is the up-regulation of enzymes/transporters that pauperize essential amino acids from the extracellular space. This results in both microenvironmental depletion of essential nutrients for T cells and in the generation of molecules endowed with immunomodulatory activities (e.g., nitric oxide, polyamines and kynurenines). Cysteine is an example of an amino acid that T cells cannot produce either by intracellular conversion of methionine or by import of extracellular oxidized cysteine (27). Usually, antigen-presenting cells couple the import of extracellular oxidized cysteine with the export of cysteine, thereby creating a circuit of symbiotic nutrients sharing that feeds T cell activation. In contrast, MDSCs up-take cystine through the xc- transporter but do not export cysteine, thus limiting the extracellular pool of cysteine required for T cell activation (28). MDSCs express copious amount of IDO1 that converts tryptophan in kinurenines inducing Treg cells expansion (29), dampening dendritic cell immunogenicity (30) and concomitantly depriving T cells of an essential nutrient (31). Several preclinical studies have demonstrated the therapeutic potential of IDO inhibition in combination with both chemotherapy and immune checkpoint blockers. Accordingly, phase II/III human trials will evaluate two small molecule enzyme inhibitors of IDO1 (epacadostat and GDC-0919/navoximod) in human cancer patients (32).

Metabolic conversion of L-arginine (L-Arg) through either iNOS or Arg1 is the first and the main mechanism associated with the immunosuppressive activities of MDSCs. In addition, Arg1 supports tumor cell proliferation by producing ornithine and polyamines, whereas iNOS promotes T cell death through NO generation and consequent tyrosine nitration and S-cysteine nitrosation of various proteins (33, 34). Strikingly, a recent paper reported that bone marrow (BM)-derived MDSCs require direct cell-cell contact rather than Arg1 expression or production of soluble factors to mediate immunosuppression in different tumor models (e. g. melanoma, colon carcinoma and lymphoma) (35).

The expression of Arg1 and iNOS differs among mouse and human myeloid cells, with the former predominantly expressed by the granulocytic subset and the latter by the monocytic counterpart (36). Preclinical studies and clinical trials with inhibitors of phosphodiesterase-5, (e.g., sildenafil and tadalafil) pointed out that a reduction of both iNOS and Arg1 activities in MDSC reactivates antitumor immunity (37–39).

Nicotinamide adenine dinucleotide (NAD) is one of the most important coenzymes in mammalian metabolic pathways (40). CD38 is an ectoenzyme that, by consuming extracellular NAD, leads to mitochondrial dysfunction of surrounding cells, as observed in metabolic diseases and cancer (41). CD38 was found up-regulated in MDSCs from various preclinical tumor models and cancer patients (neck cancer and non-small cell lung cancer). Along with the detrimental effects associated with depletion of microenvironmental NAD, CD38 generates second messengers associated with calcium signaling (42), resulting in an increased amount of NO that favors tumor growth (43). Treatment of multiple myeloma patients with daratumumab (an antibody directed toward CD38) was associated with reduction of PMN-MDSCs, suggesting that this event might contribute to the therapeutic effect of anti-CD38 (44). Beyond being a key regulator of energy metabolism and ATP production, NAD is the substrate for numerous NAD-consuming enzymes that participate in cell signaling, including mono- and poly-(ADP-ribose) polymerases, sirtuins (SIRT) and CD38/CD157 (45).

Interestingly, in different tumor models (i.e., thymoma and melanoma) the lack of SIRT1 in MDSCs fuels the glycolytic pathway through the mTOR-HIF-1α pathway. This metabolic reprogramming is associated with a functional switch of immunosuppressive MDSCs toward a pro-inflammatory (NO, TNF, IL-12) and anti-tumor phenotype (46). Additional studies confirmed the importance of metabolic pathways on MDSC activity. In both tumor bearing mice and humans, tumor-derived cytokines induce expression of cell surface lipid transport receptors on MDSCs via STAT3 and STAT5 (47). This results in increased fatty acid uptake and oxidative metabolism in association with activation of MDSCs' immunosuppressive mechanisms. Therefore, hampering the intracellular accumulation of lipids (47) as well as pharmacological inhibition of FAO (48) blocks the immunosuppressive functions of MDSCs, improving the efficacy of either immunotherapy or low-dose chemotherapy.

### Expression of Immune Checkpoint Inhibitors

It is not surprising that several pre-clinical and clinical studies have found an association between PD-L1 expression by MDSCs and immunosuppression (49). Mechanistically, the expression of PD-L1 on MDSCs can be triggered through different pathways whose relative importance may depend on different microenvironmental features of tumor regions as well as on the type of tumor. For example, hypoxia induces PD-L1 expression on MDSCs via HIF-1α (18). In line with this, the blockade of PD-L1 expression under hypoxia enables MDSCs to support T cell activation; therefore, the combination of PD-L1 neutralization with HIF-1α inhibitors could improve the clinical response of patients with advanced disease. In a preclinical model of colitis-associated colorectal cancer, PD-L1 emerged to be mainly expressed by tumor-infiltrating M-MDSCs (CD11b^+^Ly6C^+^ cells) in response to IFNγ via STAT1-IRF1 axis (49). M-CSF and VEGFA produced by human liver cancer cell lines can induce PD-L1 expression on immature myeloid cells (CD33^Dim^HLA-DR^−^ cells) isolated from peripheral blood of healthy donors. Accordingly, circulating PD-L1^+^MDSCs were detected in HCC patients and their frequency increased with disease progression, although it did not correlate with serum concentration of M-CSF or VEGF (50).

In mouse bladder cancers, PD-L1 expression on tumor-associated myeloid cells is associated with the expression of cyclooxygenase-2 (COX2), microsomal prostaglandin E synthase-1 (mPGES1), prostaglandin E2 (PGE2) and the capacity to induce apoptosis of CD8^+^ T cells (51). Either genetic or pharmacological inhibition of PGE2 restrained tumor-induced PD-L1 expression on myeloid cells. PGE2 can also directly and indirectly blunt the activation of CD8 T cells (52). Further, PGE2 has been shown to promote MDSCs activity by inducing up-regulation of additional immunosuppressive molecules (e.g., IDO, IL-10, ARG-1, and VEGF) (53–55), as well as by repressing immunogenic-associated genes via DNA methyltransferase 3A (DNMT3A) (56). In agreement, human MDSCs from ovarian cancer patients display a similar hypermethylation signature in connection with PGE2-dependent DNMT3A overexpression (56). Recently, in a pre-clinical model of colorectal cancer there has emerged a circuit based on down-regulation of receptor-interacting serine/threonine-protein kinase 3 (RIPK3) in MDSCs linked to the production of PGE2. This autocrine loop is crucial for MDSC accumulation and immunosuppressive activity and the consequent promotion of colon carcinogenesis (57). Therefore, PGE2 represents a very attractive drugable target that can be exploited to modulate MDSCs' immunosuppressive functions in multiple contexts.

Interestingly, not only are high levels of circulating MDSCs (CD33^+^CD11b^+^HLA-DR^−^cells) predictive of a poor response of advanced melanoma patients to ipilimumab (anti-CTLA4) therapy (58), but circulating MDSCs of non-responders showed higher expression of PD-L1 by PMN-MDSCs and copious production of NO by M-MDSCs (59). In line with this, in models of lung and renal cell carcinoma, entinostat, a class I histone deacetylase inhibitor, improved the anti-tumor effect of anti-PD-1 antibodies by reducing the expression of Arg1, iNOS, and COX2 in MDSCs (60). Therefore, different clinical trials are studying the combination of entinostat with immune checkpoint blockade (ICB) in patients with renal cell carcinoma and other advanced solid tumors (61).

### Production of Immunosuppressive Molecules

MDSCs express high levels of Ectonucleoside triphosphate diphosphohydrolase 1 (E-NTPDase1, CD39) and the ecto-5'-nucleotidase, which convert the extracellular ATP released by dying cells in adenosine. Extracellular adenosine is a powerful immunosuppressive factor that impairs differentiation of naïve CD8^+^ T cells in effector cells (62), inhibits cytolitic activity of NK and activated T cells (63), and it promotes the immunosuppressive functions of tumor-associated macrophages (TAM) and expansion of PMN-MDSCs (64). MDSCs also produce copious amounts of immunosuppressive cytokines, such as TGF-β and IL-10, which induce the generation of Treg cells, differentiation of pro-tumoral IL-12^low^ TAM and direct suppressive effects on T effector cells (65, 66). TGF-β can also exert either promoting or inhibiting effects on MDSCs themselves (67, 68). Exposure of murine BM-derived MDSC or healthy human PBMCs to TGF-β, along with conditioned medium of either MEER (murine pharyngeal epithelial cells expressing HPV16 E6 and E7, and hRas) or human head and neck squamous cell carcinoma cells (SCC-47), triggered CD11b^+^Gr1^+^ MDSCs to acquire antigen-presenting capability and Fas-dependent tumor cells killing activity (68). Consequently, in mice transplanted with MEER tumor, the combination of radiotherapy with intra-tumoral adoptive transfer of TGF-β-conditioned MDSCs resulted in a durable tumor clearance (68). In apparent contrast, *ex vivo* studies indicate that TGF-β skews differentiation of human peripheral blood CD14^+^ monocytes toward immunosuppressive M-MDSCs (67). Accordingly, in mouse models of lung and mammary carcinoma, disruption of TGF-β signaling in myeloid cells resulted in decreased expression of CD39 and CD73, in association with increased infiltration of T lymphocytes, reduced density of blood vessels and diminished tumor progression (69). A recent study highlighted that the frequency of CD39^+^CD73^+^ MDSCs in the NSCLC patients is closely correlated with disease progression and chemotherapeutic resistance (70). Mechanistically, it was confirmed that tumor-derived TGF-β triggers CD39 and CD73 expression on circulating and tumor-infiltrating MDSCs via activation of mTOR/HIF-1α-signaling (70). Along with these findings, diabetic patients with ovarian carcinoma gain beneficial anti-tumor effects by metformin treatment. Indeed, this anti-diabetes drug down-regulates HIF-1α via the activation of the AMP-activated protein kinase α (AMPKα) and consequently decreases expression of CD39 and CD73 on both M- and PMN-MDSCs. Therefore, metformin treatment leads to the reduction of circulating CD39^+^CD73^+^ MDSCs and enhances the anti-tumor activities of circulating CD8^+^ T cells, promoting longer overall survival of ovarian cancer patients (71). New evidence indicates that MDSCs can secrete exosomes which contain molecules, such as immunosuppressive Arg-1 (72), inflammatory S100A8/9 (73) and the oncogenic miR-126a (74). Interestingly, *in vivo* administration of PMN-MDSCs derived exosomes to DSS-treated mice ameliorates colitis, thereby confirming the immunosuppressive activity of molecules included in the extracellular vesicles (EV) (72). In cancer bearers, tumor cells are the major source of circulating EV. Recently a set of microRNAs (miR-146a, miR-155, miR-125b, miR-100, let-7e, miR-125a, miR-146b, miR-99b) has been identified that are transferred via EV from melanoma cells to circulating monocytes, driving their conversion into MDSCs. Therefore, high levels of plasma MDSC-miRs emerged as valuable predictive peripheral blood biomarkers of resistance to ICB in cancer (75).

### Production of ROS

A major mechanism used by PMN-MDSCs to suppress antigen-specific T cells is the secretion of copious amounts of reactive oxygen species (ROS), including superoxide anions, hydroxyl radicals, hydrogen peroxide and singlet oxygen (34). Accordingly, in a MDSCs/T cells co-culture system, the addition of ROS inhibitor catalase blunts the immunosuppressive effects of MDSCs (76). ROS production by MDSCs is driven by the up-regulation of NADPH oxidase activity, in particular the NOX2 subunits 47 (phox) and gp91 (phox). Indeed, the lack of NOX2 impaired both generation of ROS by MDSCs and their ability to suppress antigen-specific CD8^+^ T cells (77). In addition, NOX2-dependent ROS production supports MDSC expansion (77) and recruitment in tumors through the up-regulation of VEGF receptors (78). Myeloperoxidase is another ROS-producing enzyme that, along with ARG-1, is more abundantly expressed by PMN-MDSCs than neutrophils, contributing to suppression of antigen-specific T cell responses in tumor bearers (79).

MDSCs survive despite elevated levels and continuous production of ROS through the expression of the Nrf2 transcription factor, an important mediator of the cellular antioxidant response (80). Indeed, genetic ablation of Nrf2 impaired generation, survival and suppressive potency of MDSCs in models of mammary and colon tumor (80). To counteract the detrimental effects of oxidative stress, MDSCs up-regulate their anaerobic metabolism (i.e., glycolysis), which leads to the intracellular accumulation of the anti-oxidative intermediate phosphoenolpyruvate (81). Overall, targeting redox-regulation of MDSCs is emerging as a promising therapeutic opportunity in multiple diseases, such as cancer, infection, inflammation, and autoimmune disorders (82).

### Regulation of Lymphocyte Homing

MDSCs impair T cell activation also by inhibiting the homing of naïve CD4^+^ and CD8^+^ T cells to lymph node (83). This effect is dependent on down-regulation of CD62L on naïve T cells through the expression of TNF-α-converting enzyme (TACE/ADAM17) by MDSCs (84). Growing evidence suggests that this ability of MDSCs to hinder T cell activation plays a crucial role in establishing maternal–fetal tolerance in pregnant mice and women (85). An expansion of MDSCs in maternal peripheral blood occurs during human pregnancy, and higher frequency of circulating PMN-MDSCs has recently emerged as a favorable predictor of the success rate of *in vitro* fertilization treatment (86). In addition, MDSCs can hamper the recruitment of circulating effector T cells into tissues by inhibiting the expression of CD162, a ligand of P-selectin and CD44, the receptor for the extracellular matrix component hyaluronic acid (HA) (87). The block of effector T cell homing is paralleled with the recruitment of immunosuppressive T cells. For example, in two mouse models of melanoma, tumor M-MDSCs produce CCL3, CCL4 and CCL5 which drive the recruitment of CCR5^+^ Treg cells (88).

## MDSCs in Autoimmunity

Autoimmunity is defined as an immune response against self-antigen. The tolerance against self-antigens is a tightly regulated process that involves both innate and adaptive immunity and implies the possibility to eliminate or inhibit self-reactive lymphocytes. In autoimmune diseases (AD), both genetic and environmental factors contribute to the breakdown of tolerance (89), which results in the generation of auto-reactive B and T cells. Clinical manifestation of AD derives from tissue damage caused by self-reactive T cells. In contrast to their deleterious role in tumors, MDSCs have been studied in various models of AD to evaluate potential beneficial role (90).

Due to their prevalent immunoregulatory phenotype MDSCs represent an important cell population that can be therapeutically used to suppress T cell functions. On this line, new work indicates their accumulation in secondary lymphoid organs of patients with autoimmune disorders, including type 1 diabetes, multiple sclerosis, rheumatoid arthritis, systemic lupus erythematosus, inflammatory bowel disease and autoimmune hepatitis (91), and a number of studies have provided insight into the use of MDSCs for treatment of AD (12).

### Autoimmune Diabetes

Type 1 diabetes (T1D) is among the most prevalent autoimmune diseases worldwide, affecting ~10–20 million people. The disease occurs as a consequence of a disruption in immune-regulation, resulting in the expansion of autoreactive CD4 and CD8 T cells and autoantibody-producing B lymphocytes (92), which leads to the destruction of pancreatic insulin-producing β-cells in the pancreas (93). Both CD4^+^ helper T cells and CD8^+^ cytotoxic T cells can transfer autoimmune diabetes to immunodeficient hosts in mouse models (94, 95), and T cells are found in inflammatory infiltrates surrounding pancreatic islets in T1D patients (96).

Rising evidence of MDSCs' involvement in the pathogenesis of T1D opens new potential therapeutic strategies for T1D. In two different murine models, Yin et al. provided evidence that adoptive transfer of MDSCs against autoreactive T cells prevented pancreatic islets damage (97). Furthermore, it was shown in NOD/SCID mice that temporary B cell depletion induced expansion of regulatory CD11b^+^Gr1^+^ cells, which directly suppress diabetogenic splenocytic T cell functions in an IL-10-, NO-, cell contact-dependent manner (98).

It has also been shown that the contribution of the C3 complement factor to the development of autoimmune diabetes depends directly on MDSCs. In fact, the C3 deficiency in the Streptozotocin-induced diabetes (STZ) model produces an increase in the frequency of MDSCs and enhances their ability to suppress the proliferation of diabetogenic T cells through arginase/iNOS activity (99).

A paradoxical increase in the frequency of MDSCs was reported in the peripheral blood of T1D patients, as well as in the peripheral blood and secondary lymphoid organs of diabetic NOD mice (100). Of note, this increased frequency of MDSC was counterbalanced by the decreased MDSC number within the pancreatic microenvironment of diabetic NOD mice, suggesting that the lack of islet MDSCs may favor autoimmune diabetes development (100).

A strong association has been demonstrated between polymorphisms of NOD-like receptor family-pyrin domain containing 3 (NLRP3) and predisposition to the T1D. Carlos et al. showed that the ablation of NLR3P in both NOD and STZ-treated diabetic mice, as a consequence of elevated IL-6 expression, produced an expansion of MDSCs in pancreatic lymph nodes (PNLs), which inhibits the inflammatory T cells response in the pancreatic islets and prevents the onset of T1D (101). This evidence proposes the expansion of MDSCs as strategy for dampening the autoimmune T cell response and preventing T1D.

### Multiple Sclerosis (MS)

MS is an autoimmune inflammatory demyelinating disease and a prime cause of neurological disability in young adults (102). Clinically, MS manifests itself as neurological deficits that frequently exhibit a relapsing and remitting pattern (RRMS) reflecting the characteristic recurrent bouts of T cell-mediated attack upon antigens in neuronal myelin sheaths. MS can resolve completely or leave residual deficits of any grade (102).

Experimental Autoimmune Encephalomyelitis (EAE) is the most used animal model of autoimmune inflammatory diseases of the central nervous system (CNS), and it resembles MS. Active EAE is induced by immunization with CNS tissue or myelin peptides, such as myelin basic protein (MBP) and proteolipid protein (PLP) emulsified in various adjuvants, usually containing bacterial components highly capable of activating the innate immune system via pattern recognition receptors (i.e., complete Freund's adjuvant, CFA) (103). This leads to the peripheral activation of myelin-specific T cells which are subsequently recruited together with myeloid cells in the CNS. These provoke the release of inflammatory cytokines and chemokines, producing demyelination and CNS damage (103, 104).

In the last decade, the presence and the activation state of MDSC subsets in MS have been objects of intense investigation. In a model of experimental autoimmune encephalomyelitis (EAE), Zhu et al. first characterized the subsets of accumulating myeloid cells in blood, spleen and CNS. They showed that a small population of CD11b^+^Ly6C^hi^ immature monocytic cells could exert the potent suppression of both CD4^+^ T cells and CD8^+^ T cells *ex vivo*, inducing their apoptosis through nitric oxide production (105).

In contrast, two different and independent works highlighted a more pro-inflammatory and pathogenetic role of the CD11b^+^Ly6C^hi^ cell subset. Mildner et al. proposed that CCR2-expressing CD11b^+^Ly6C^hi^ monocytes are indispensable for the pathogenesis of MS due their capability to express MHC class II molecules and inflammatory cytokines, which would support local autoimmune encephalitogenic T cell activation (106).

King et al. instead proposed a dynamic interpretation of the role of CD11b^+^Ly6C^hi^ cells in MS. CD11b^+^Ly6C^hi^ cells that accumulate in the blood and CNS of mice immunized by myelin, before the onset of clinical episodes, would behave like inflammatory monocytes rather than MDSCs. Next, the CNS microenvironment would evolve during the course of the disease, inducing a more suppressive and anti-inflammatory phenotype of CD11b^+^Ly6C^hi^ immediately before the onset of the remission phase (107). In line with this, the distribution of protective Arg1-expressing MDSCs within the spinal cord of EAE mice was confirmed during the remitting phase (108).

A pivotal role in the regulation of CNS autoimmune inflammation was provided also for PMN-MDSCs, which accumulate in the peripheral draining lymph nodes (LNs) and in the spinal cord of EAE-immunized mice, prior to the remission phase of the disease (109). Moreover, granulocytic CD33^+^CD15^+^ MDSCs were significantly enriched in the peripheral blood of subjects with active MS (109). Noteworthily, in EAE mice, adoptively transferred PMN-MDSCs ameliorated the disease and delayed its onset through the significantly reduced expansion of autoreactive T cells in the draining of LNs (109).

The initiation and severity of the chronic disease phase in MS is associated with the accumulation of these B cell aggregates. Knier et al. showed that the frequency of CD138^+^ B cells in the cerebrospinal fluid (CSF) of human patients with MS was negatively correlated with the frequency of PMN-MDSCs in the CSF (110).

Analyses of the dynamic of immune cell populations in the CSF and CNS parenchyma of mice during EAE revealed a persistent population of Ly6G^+^ cells recruited to the CSF space at the beginning of the recovery stage. Cantoni et al. have recently identified that the decreased number of blood M-MDSCs in relapsing MS patients is associated with increased MDSC expression of miR-223 compared to healthy subjects, and it is accompanied by a reduced expression of STAT3 and Arg1 (111, 112). These data are corroborated by the evidence that miR-223 deficient mice showed reduced EAE severity and pathology progression as result of an increase in MDSC number in the spleen and CNS (112).

### Rheumatoid Arthritis (RA)

RA is a systemic AD characterized by a chronic synovitis that results from the sustained influx of various leukocyte populations into the synovial space, thereby leading to destruction of the joint cartilage and erosion of bone (113). CD4^+^ T cells, and the cytokine milieu within the affected joints, are critically implicated in the pathogenesis of RA, as they promote differentiation toward pro- and anti-inflammatory T cell subpopulations, including Th1, Th2, Th17, and Treg cells (114). Elevated levels of pro-inflammatory Th17 cells as well as defects in anti-inflammatory Treg cells have been reported in RA patients (115, 116) and in experimental arthritis in mice (117, 118), but the mechanisms governing the imbalance of Th17/Treg cells resulting in RA remain unclear. Discordant results regarding the effect of MDSCs on RA have been reported in both preclinical mouse models and patients (119). Jiao et al. reported that both the prevalence of circulating MDSCs and plasma Arg1 increased significantly in RA patients compared to healthy controls and were negatively correlated with peripheral Th17 cells (116). Unfortunately, these MDSC-like cells were defined only by phenotypic marker expression, and the suppressive properties of these cells toward T cells were not tested in that study. A beneficial accumulation of MDSCs, mainly PMN-MDSCs, was reported in the spleens of arthritic DAB/1 mice with collagen-induced arthritis (CIA) at the peak of the disease (35 days after CIA induction), and these cells prevented both the proliferation of CD4^+^ T cells and their differentiation into Th17 cells *in vitro*, via an Arg1-dependent mechanism (120). Moreover, *in vivo* depletion of PMN-MDSCs with anti-Gr1 mAb delayed the spontaneous resolution of joint inflammation in mice with CIA, while adoptive transfer of CD11b^+^Gr1^+^ MDSCs reduced the severity of CIA *in vivo* and decreased the number of total CD4^+^ T cells and Th17 cells in the dLN (120). It has also been demonstrated that PMN-MDSCs in synovial fluid (SF) from mice with proteoglycan (PG)-induced arthritis (PGIA) could potently suppress autoreactive T cell proliferation and dendritic cell maturation (121). Recently, the adoptive transfer using three types of splenic MDSCs (total MDSCs, M-MDSCs and PMN-MDSCs) obtained from CIA mice demonstrated that all these kinds of MDSCs markedly ameliorated inflammatory arthritis and profoundly inhibited T cell proliferation (122). All the aforementioned studies revealed promising therapeutic effects of MDSCs in an animal model of RA (120–122). However, a few recent papers have shown that MDSCs can aggravate inflammatory arthritis in mice (123–125). Such a discrepancy in the results could be due to the heterogeneity of MDSCs, inflammatory context-dependent interaction between MDSCs and different subsets of CD4^+^ T cells and different states of disease.

### Systemic Lupus Erythematosus (SLE)

Lately, the possible involvement of MDSCs in SLE, a systemic AD characterized by elevated levels of autoantibodies against nuclear materials (ANAs) and cellular infiltration of various organs (126), has also been addressed. Administration of laquinimod, an immunomodulatory drug currently in clinical trials for MS and lupus nephritis, in a (NZB × NZW)F1 murine model of SLE, delayed lupus manifestation by inducing expansion of M-MDSCs and PMN-MDSCs in the spleen and kidney (127). In addition, IL-33 blockade in MRL/Fas^lpr^ mice could significantly ameliorate the severity of SLE disease, and this therapeutic effect was closely associated with expansion of MDSCs and Treg cells, accompanied by reduced Th17 cells and inflammatory cytokines in the serum and kidneys (128). Another study reported that deletion of CD24 in a lupus-like disease model (tm24KO mice) led to the expansion of MDSCs and Treg cells that augmented immune tolerance, accompanied with the alleviation of lupus-like pathology (129).

However, the protective role of MDSCs in lupus is challenged by much evidence. A significant increase in HLA-DR^−^CD11b^+^CD33^+^ MDSCs, including both CD14^+^CD66b^−^ monocytic and CD14^−^CD66b^+^ granulocytic MDSCs, was reported in the peripheral blood of patients with active SLE, and the frequency of these populations positively correlated with serum Arg1 activity, Th17 responses and lupus severity. Moreover, adoptive transfer of non-MDSC-depleted PBMCs from SLE patients in NOD/SCID mice, induced lupus nephritis-like symptoms via Th17 response in an Arg1-dependent manner (130). A critical pathogenic role of MDSCs was recently documented also in lupus nephritis (LN), one of the most severe manifestations of SLE. In particular, in a TLR-7 agonist imiquimod-induced lupus mice model, MDSCs induced severe podocyte injury in the glomeruli of kidneys through increasing ROS, activating p-38MAPK and NF-κB signaling (131). These data infer that changes in both percentage and function of MDSCs could be crucial during SLE development; however, it is still not clarified which factors influence the behavior of MDSCs in the lupus microenvironment.

### Inflammatory Bowel Disease (IBD*)*

In IBD an aberrant homeostasis between intraluminal bacterial antigens and the mucosal immune system leads to chronic inflammatory pathology. IBD encompasses both Crohn's disease (affects any part of the gastrointestinal part) and ulcerative colitis (affects colon and/or rectum) (132, 133). IBD is widely considered to result from an overlay aggressive Th1 immune response and excessive IL-23/Th17 pathway activation, as well as decreased Treg responses (134). Interestingly, an increase in the frequency of human CD14^+^HLA-DR^−/low^ MDSCs with suppressive properties was observed in the peripheral blood from IBD patients (135, 136). In agreement, hyperactivation of STAT3, a known regulator of MDSC expansion, has been associated recently with protection from experimental colitis (137, 138), while another study reported that the resistance to colitis in gp130^757F/F^ mice occurred via myeloid-specific STAT3 activation, expansion of PMN-MDSCs in the colon and increased production of suppressive cytokines (138). In contrast with these observations, another study showed that adoptively transferred BM Ly6C^high^ cells are recruited into the colon and differentiate into inflammatory DCs and macrophages (139), contributing to intestinal inflammation in a TNFα-dependent manner (140) and triggering proliferation of antigen-specific T cells (141). In addition, a recent paper reported that IBD patients had high peripheral blood levels of CD14^+^HLA-DR^−/low^ MDSCs, associated with exacerbated IBD (142). Hence, the intrinsic plasticity of MDSCs renders them prone to conversion into effector cells; it is very important to evaluate how their suppressive potential can be harnessed therapeutically to benefit IBD patients.

### Others (Myastenia, Psoriasis, Uveitis, Trombocytopenia)

Additional evidence supporting the immune regulation capabilities of myeloid cells in ADs came from a mouse model of myasthenia gravis, by which McIntosh and Drachman showed a population of “large suppressive macrophages” (LSM) capable of suppressing T cell proliferation (143). A counterintuitive role of MDSCs is emerging in psoriasis. Psoriatic patients display an increased frequency of granulocytic and monocytic MDSC subtypes in blood and skin compared to healthy subjects (144–146). Lauren et al. highlighted a high heterogeneity of MDSCs in this pathology in terms of a diverse capability to inhibit allogeneic T cells through the use of either the IL-17/Arg-1 or IFNγ/iNOS axis as suppressor mechanisms (144). Furthermore, these cells are capable of producing various molecules, including matrix metalloproteinase-9 and−1, interleukin-8, growth-related oncogene, and monocyte chemoattractant protein 1, which could contribute to further establishing a pro-inflammatory immune response and confer less immunosuppressive attitudes to MDSCs (145). Soler et al. showed that psoriatic M-MDSCs directly suppressed CD8^+^ T-cell proliferation less efficiently than healthy control M-MDSCs (146). Kerr et al. have also described a dynamic presence of MDSCs in the inflamed eye of autoimmune uveoretinitis (EAU) subjects. In this model, MDSCs isolated from the inflamed eye were able to profoundly suppress T cell proliferation (147). In another study, this group showed an infiltrating subset of CD11b^+^Gr1^+^Ly6C^+^ cells which suppressed the T cell mediated pro-inflammatory response in a TNF receptor 1-dependent manner (148). Finally, Hou et al. have described impaired numbers and suppressive functions of MDSCs in the blood and spleens of adult patients with primary immune thrombocytopenia (ITP), where cell-mediated immune responses are involved in platelet destruction (149). The overall scenario indicates that MDSC manipulation may provide therapeutic benefit during the course of autoimmune disorders.

### Perspectives of MDSC Reprogramming in Therapy

The gold-standard treatment for autoimmune diseases relies on immunosuppressive drugs because of their high effectiveness in ameliorating symptoms in many patients. However, long-term and high-dose administration of such drugs can lead to life-threatening, opportunistic infections and long-term risk of malignancy (150). Furthermore, the generation of new therapeutic approaches exploiting the CTLA-4-mediated costimulatory blockade (151–154) or the neutralization of pro-inflammatory cytokines (155) frequently result in increased side effects and lack of responsiveness in long-term administration. In this scenario, cell-based therapy that exploits the *ex vivo* generation of MDSCs represents an interesting perspective for the treatment of ADs. Indeed, compelling evidence from animal models has provided insights into the potential therapeutic effects of MDSCs adoptive transfer in ADs.

In a murine model of arthritis, BM progenitor cells of healthy mice cultured with a combination of IL-6, G-CSF and GM-CSF became enriched in MDSC-like cells that potently inhibited antigen-specific and polyclonal T-cells proliferation *in vitro* via the production of nitric oxide. The injection of BM-MDSCs into mice with PGIA ameliorated arthritis and reduced PG-specific T cell responses and serum antibody levels (156). Moreover, addition of tofacitinib (a small-molecule JAK inhibitor currently considered as novel therapy of RA) facilitated the *in vitro* expansion of MDSCs inhibiting their differentiation to DCs, and their adoptive transfer in SKG arthritic mice reduced the severity of the disease (157). A therapeutic effect of BM-derived MDSCs was demonstrated also in a model of SLE. Intravenous injection of MDSCs, differentiated from BM cells of C57BL/6 mice upon stimulation with M-CSF and GM-CSF, induced expansion of Breg cells via iNOS and ameliorated autoimmunity in Roquin^san/san^ lupus mice (158). In another study, BM cells were isolated from wt mice and cultured in the presence of GM-CSF and HSCs, resulting in the generation of MDSCs. Adoptive transfer of these MDSCs in mice with colitis, induced by 2,4,6-trinitrobenzenesulfonic acid (TNBS), decreased intestinal inflammation as well as the levels of IFNγ, IL-17 and TNFα (159). Likewise, in the study by Su et al., which investigated the role of MDSCs in the model of TNBS-induced colitis, transplantation of GM-CSF-induced MDSCs ameliorated intestinal inflammation and downregulated the levels of proinflammatory cytokines (160). In the STZ-treated diabetic mice, Hsieh et al. provided proof that the adoptive transfer of MDSCs, obtained from BM cells cultured *in vitro* with GM-CSF, IL-1β, and IL-6 under normoglycemic conditions, substantially reduced fibronectin accumulation in the renal glomerulus, ameliorating diabetic nephropathy (161). In a mouse model of alopecia areata (AA), it was recently demonstrated that MDSCs can efficiently exert their activity not only through cell-cell contacts or soluble factors, but also by their capability to secret exosomes (Exo) (162). Indeed, Zöller et al. showed that treatment with MDSC-derived Exo from naïve mice prevented the progression of the disease and induced partial hair growth as a result of the inactivation of pro-inflammatory T cells and promotion of T regulatory cell differentiation (163).

Other potential opportunities of MDSC-mediated cell therapy apply to allogeneic transplantation. In this regard, Highfill et al. showed that addition of IL-13 in BM cells cultured with GM-CSF and G-CSF resulted in the production of suppressive MDSCs that efficiently inhibited allo-immune rejection (164). In pancreatic islet transplantation, Chou et al. observed that the presence of small amounts of Hepatic stellate cells (HpSC) into DC culture (BM-cells stimulated for 5 days with GM-CSF) produced a large number of MDSCs that efficiently protected islet allografts (165). Importantly, in this model of allograft transplantation, as well as in transplantation of male skin onto female recipients, it was found that only long-term and multiple injections of MDSCs significantly improved the acceptance of the graft (166). This may be due to the observation that in absence of chronic inflammation MDSCs may terminally differentiate toward a pro-inflammatory phenotype (167, 168). This evidence highlights the need to identify new strategies that stabilize the suppressive phenotype of MDSCs. In this regard, Greifenberg et al. showed that BM-MDSCs differentiated in the presence of LPS and IFN-γ expressed a stable suppressive phenotype (169). Therefore, although many open questions on the therapeutic use of MDSCs remain to be clarified, an increasing number of observations indicate that these cells can potentially be used to control autoimmune diseases and allograft rejection.

## Discussion

**Figure 2:**
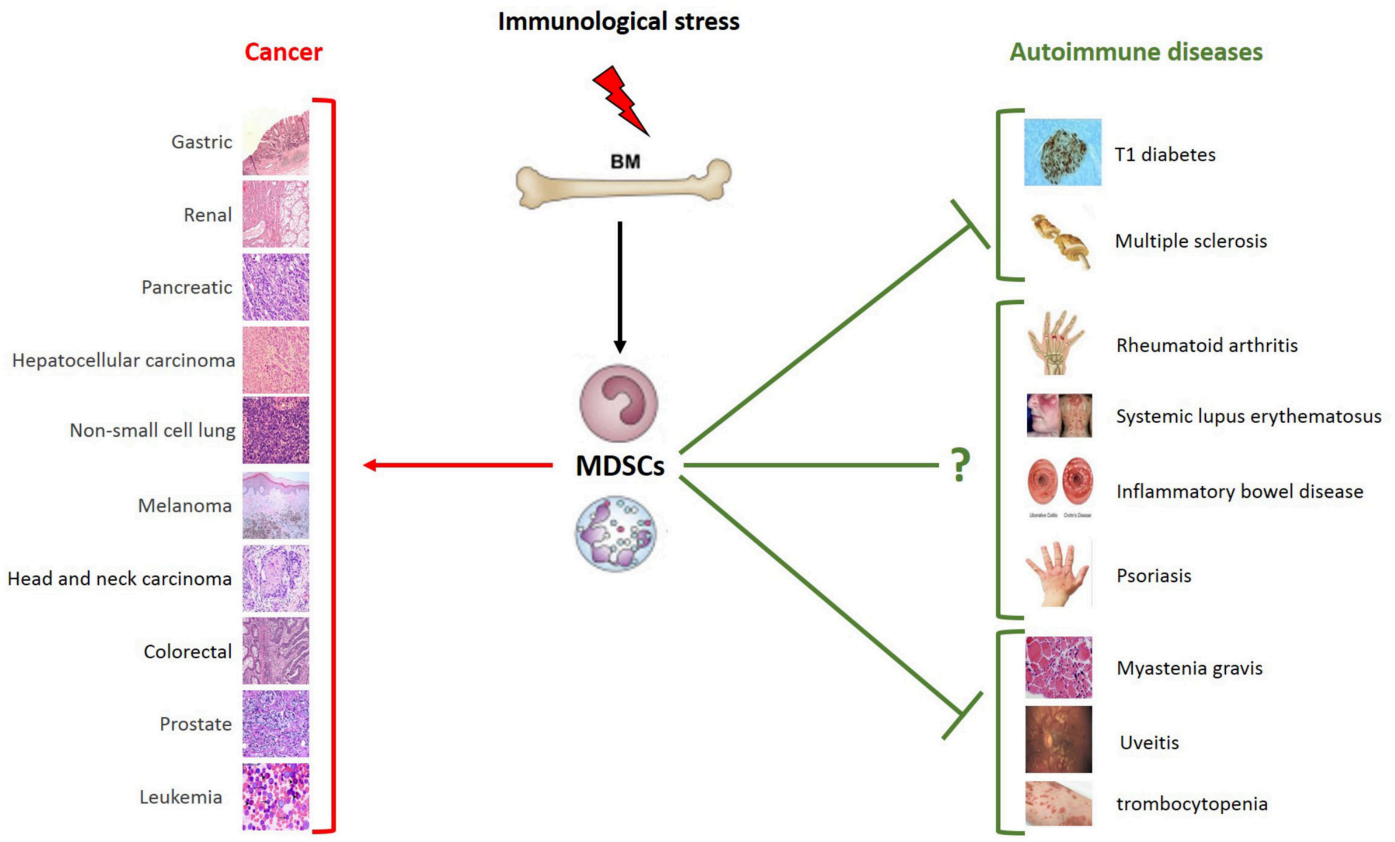
Schematic role of MDSCs in pathology. Immunological stress induces the expansion of MDSCs that play different roles depending on distinct pathological and microenvironmental contexts. MDSCs are characterized by the strong ability to suppress T cell functions. Much clinical and preclinical evidence demonstrates their ability to promote tumor growth and metastasis formation. Given the immunosuppressive phenotype, MDSCs can also play a beneficial role in autoimmune diseases. As shown in the figure, the expansion of MDSCs is protective in some autoimmune diseases, such as type 1 diabetes, multiple sclerosis, myastenia gravis, uveitis and trombocytopenia. Their role in systemic lupus erythematosus, inflammatory bowel disease psoriasis and rheumatoid arthritis remains to be further clarified. The types of tumor and autoimmune diseases in which an expansion of MSDCs have been reported are summarized in the figure. MDSCs, myeloid-derived suppressor cells; BM, bone marrow.

MDSCs violently emerge in pathological conditions in an attempt to limit potentially harmful immune and inflammatory responses. Mechanisms supporting their expansion and survival are deeply investigated in cancer, in the perspective to reactivate specific antitumor responses and prevent their contribution to disease evolution. These findings will likely contribute to improve the targeting of MDSCs in anticancer immunotherapies, either alone or in combination with immune checkpoint inhibitors. New evidence indicates that the expansion of myeloid cell differentiation in pathology is subject to fine-tuning, as its alterations may support either immunosuppression or autoimmunity. This pathological plasticity is supported by evidence indicating that common MDSC-associated targets may be specularly targeted in autoimmunity vs. cancer (12), and there is now hope that understanding autoimmune mechanisms might serve as a lesson for the development of new anticancer therapies. The functional plasticity and therapeutic ductility of these cells (Figure 2) suggest that while MDSC inhibition might succeed as anticancer treatment, their induction is expected to provide therapeutic benefit in autoimmune diseases.

## Author Contributions

All authors listed have made a substantial, direct and intellectual contribution to the work, and approved it for publication.

### Conflict of Interest Statement

The authors declare that the research was conducted in the absence of any commercial or financial relationships that could be construed as a potential conflict of interest.
